# Targeting Vascular NADPH Oxidase 1 Blocks Tumor Angiogenesis through a PPARα Mediated Mechanism

**DOI:** 10.1371/journal.pone.0014665

**Published:** 2011-02-07

**Authors:** Sarah Garrido-Urbani, Stephane Jemelin, Christine Deffert, Stéphanie Carnesecchi, Olivier Basset, Cédric Szyndralewiez, Freddy Heitz, Patrick Page, Xavier Montet, Liliane Michalik, Jack Arbiser, Curzio Rüegg, Karl Heinz Krause, Beat Imhof

**Affiliations:** 1 Department of Pathology and Immunology, Centre Médical Universitaire, University of Geneva, Geneva, Switzerland; 2 Department of Pediatrics, Centre Médical Universitaire, University of Geneva, Geneva, Switzerland; 3 GenKyoTex S.A., Geneva, Switzerland; 4 Department of Physiology and Metabolism, Centre Médical Universitaire, University of Geneva, Geneva, Switzerland; 5 Center for Integrative Genomics, University of Lausanne, Lausanne, Switzerland; 6 Department of Dermatology, Emory University School of Medicine, Atlanta, Georgia, United States of America; 7 Department of Medicine, Faculty of Science, University of Fribourg, Fribourg, Switzerland; Universität Heidelberg, Germany

## Abstract

Reactive oxygen species, ROS, are regulators of endothelial cell migration, proliferation and survival, events critically involved in angiogenesis. Different isoforms of ROS-generating NOX enzymes are expressed in the vasculature and provide distinct signaling cues through differential localization and activation. We show that mice deficient in NOX1, but not NOX2 or NOX4, have impaired angiogenesis. NOX1 expression and activity is increased in primary mouse and human endothelial cells upon angiogenic stimulation. NOX1 silencing decreases endothelial cell migration and tube-like structure formation, through the inhibition of PPARα, a regulator of NF-κB. Administration of a novel NOX-specific inhibitor reduced angiogenesis and tumor growth in vivo in a PPARα dependent manner. In conclusion, vascular NOX1 is a critical mediator of angiogenesis and an attractive target for anti-angiogenic therapies.

## Introduction

Angiogenesis is a complex process occurring in physiological situations such as embryogenesis and wound repair, and contributes to pathological conditions such as diabetes, psoriasis, arthritis and cancer. Angiogenesis is a critical determinant of cancer progression. In its absence, tumors are unable to grow beyond the size of microscopic lesions and persist as dormant, non-expanding nodules [1], [2]. Tumor cells, stromal cells and infiltrating bone marrow-derived cells can initiate angiogenesis through a process called angiogenic switch in which secretion of pro-angiogenic factors is increased and/or production of endogenous anti-angiogenic factors is reduced [3], [4]. Angiogenic vessels are mostly formed by sprouting of endothelial cells from the existing vasculature. This process involves degradation of the surrounding matrix, cell proliferation, migration, differentiation, and tube formation [5]. Inhibition of angiogenesis has recently been introduced in the clinics as novel therapeutic option to block cancer progression [6].

NADPH oxidases are enzymes that produce reactive oxygen species (ROS). Depending on concentration and sub-cellular localization, ROS can mediate a variety of cellular functions, including pathogen killing, cell migration, proliferation and differentiation (for review [7]). The NADPH oxidase (NOX) family of proteins consists of seven isoforms (NOX1-5 and DUOX 1-2), which transport electrons across membranes, thereby reducing oxygen into superoxide. Depending on the isoform, these catalytic transmembrane proteins form a complex with p22^phox^ and the cytoplasmic subunits p67^phox^/NOXO1, p47^phox^/NOXA1, p40^phox^ and Rac1/2 [7]. The NOX1 isoform is expressed in epithelial cells, retinal pericytes, osteoclasts, vascular smooth muscle and endothelial cells [8]–[16]. While increased NOX1 expression has been reported in cases of colon cancer [17], it has been suggested that this may correlate with inflammation rather than tumorigenesis [18], [19]. However, experimental overexpression of NOX1 in fibroblasts or carcinoma cells induced an angiogenic switch mediated by the increased production of VEGF and MMPs [20]. Moreover NOX1 has been shown to regulate apoptosis and morphogenesis of sinusoid endothelial cells in vitro [16].

The nuclear hormone receptors peroxisome proliferator-activated receptors (PPAR) dimerize with the retinoid X receptor. Upon activation by lipids, this complex regulates gene transcription by binding to peroxisome proliferator-responsive elements. PPARα, a member of the family, was shown to mediate anti-inflammatory activity through inhibition of the transcription factor NF-κB. In the vascular system, PPARα inhibits NF-κB transactivation either by direct interaction with the p65 subunit or by up-regulation of I-κB, the NF-κB inhibitory subunit [21], [22]. In different tumor models, activation of PPARα by agonists blocks tumor growth and angiogenesis by reducing production of proangiogenic factors such as VEGF or epoxyeicosatrienoic acids [23]–[25]. In human endothelial cells, PPARα activators also inhibit cytokine-induced expression of adhesion molecules and chemokines [26].

In this study, we analyzed the role of NOX1 in human and mouse angiogenesis and observed an increased expression and activity of NOX1 during the angiogenic switch. Furthermore, blood vessel formation in NOX1-deficient mice was dramatically reduced in response to angiogenic factors and in tumors. NOX1 deficiency also lead to reduced endothelial cell migration and reduced formation of tube-like structures. We analyzed the mechanism by which NOX1 regulates angiogenesis and showed that NOX1 down-regulates expression and activity of the anti-inflammatory and anti-angiogenic nuclear receptor PPARα.

## Results

### NOX1-deficient mice show impaired angiogenesis

**Figure 1:**
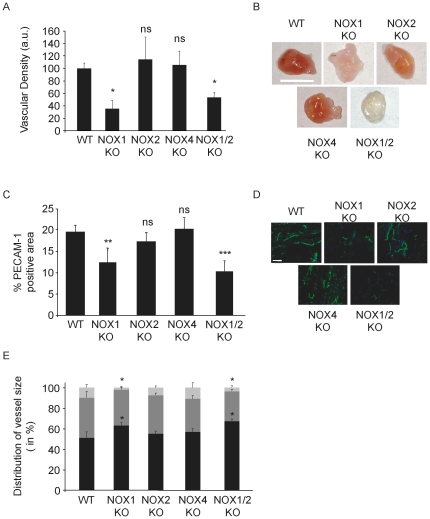
NOX1 deficient mice exhibit reduced angiogenic capacity. Matrigel was loaded with 500 ng/ml bFGF and implanted subcutaneously into NOX deficient mice. After one week, iodinated liposomes were injected i.v. and the plugs analyzed by X-ray tomography. (a) Quantification of matrigel plug vascularization. Graph shows mean of grey density ± s.e.m. For WT, NOX1 KO and NOX1/2 KO n = 8 and for NOX2 KO and NOX4 KO n = 6. (b) Photographs of excised plugs, scale bars represent 1 cm. c. Blood vessel density in plugs from the experiment in (a). Graph shows percentage of PECAM-1 positive area ± s.e.m. (d). Photographs of PECAM-1 immunostaining, PECAM-1 in green, nuclei in blue, scale bars represent 20 µm. Images were acquired with a 20x/0.5 numeric aperture lens and analyzed using LSM510 Meta confocal microscope (Carl Zeiss). e. Vessel size analysis; vessel with lumen under 20 µm are considered as small (black), from 20 to 50 medium (dark grey) and over 50 µm as large (light grey) ± s.e.m. Anova p<0.01; * p<0.05, ** p<0.01, *** p<0.001.

In order to test whether NOX-dependent ROS production participates in blood vessel formation, we performed *in vivo* Matrigel angiogenesis assays using mice deficient for different NOX isoforms. Matrigel was preloaded with the angiogenic factor bFGF and implanted subcutaneously into wild type (WT) or NOX-deficient mice. Subcutaneous Matrigel plug vascularization quantified by X-Ray-based computer tomography in animals deficient for NOX2 or NOX4 had angiogenic responses indistinguishable from WT mice. By contrast, Matrigel plug vascularization was reduced by 47% ±7.6 and 65% ±13.2 in NOX1 knockout and NOX1/2 double knockout animals respectively, as compared to WT mice (Figure 1a). This difference is noticeable on macroscopic images of Matrigel plugs following excision (Figure 1b). Immunostaining of plugs with the vascular marker PECAM-1 demonstrated reduced PECAM-1-positive areas in NOX1-and NOX1/2-deficient mice (56% ±2 and 46% ±3 respectively) compared to WT animals (Figure 1c,d). Remarkably, plugs in NOX1-and NOX1/2-deficient mice were lacking large vessels while the number of small vessels significantly increased (Figure 1e).

From these results, we conclude that NOX1 is essential for bFGF-induced angiogenesis.

### NOX1 expression and activity are up-regulated by proangiogenic factors

**Figure 2:**
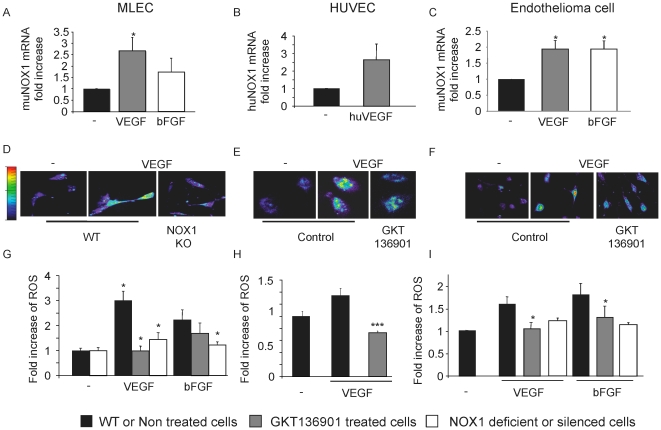
NOX1 expression and activity is up-regulated by angiogenic stimuli. NOX1 expression was measured by quantitative real-time PCR in mouse primary lung endothelial cells (MLEC, a), HUVEC (b), mouse endothelioma cell line (c) after stimulation with VEGF or b-FGF at 20 ng/ml for 3 hours. The quantity of NOX1 mRNA was normalized to the quantity of a housekeeping gene, tubulin for mouse cells and β2-microglobulin for human cells, ± s.e.m, n = 3. Activity of NOX1 was evaluated by ROS measurement using DHE substrate on MLEC (d,g), HUVEC (e, h) and mouse endothelioma (f, i). ROS production is up-regulated by VEGF and b-FGF stimulation in a NOX1-dependent manner. g, h, i. Graphs show quantification of ROS level in endothelial cells stimulated in presence or absence of VEGF or b-FGF. g. MLEC WT (black bar), MLEC WT + GKT136901 (grey bar), MLEC NOX1KO (white bar). h. HUVEC untreated (black bar) or treated with GKT136901 at 10 µM (grey bar). i. Mouse endothelioma cell line untreated (black bar), treated with GKT136901 at 10 µM (grey bar), treated with NOX1 siRNA (white bar). Results are expressed in fold increase ± s.e.m, n = 3. *p<0.05, ***<0.001 using Student's t-test.

To determine whether the aberrant angiogenesis observed in NOX1-deficient mice was due to impaired endothelial cell function; we studied the effect of NOX1 inhibition on mouse lung endothelial cells (MLEC) [27] (Figure S1), murine thymic endothelioma (tEnd) cells and primary human umbilical vein-derived endothelial cells (HUVEC). We first studied the expression level of NOX1 in these cells under basal conditions and in response to stimulation with the angiogenic factors VEGF or bFGF at 20 ng/ml for 3 hours. Quantitative real-time PCR revealed 2–3 fold up-regulation of NOX1 mRNA expression in all endothelial cells after angiogenic stimulation (Figure 2a–c). Expression of NOX4 and NOX2 did not change upon VEGF or bFGF stimulation (data not shown). While endothelial NOX4 expression level was high, NOX2 was found to be very low.

**Figure 3:**
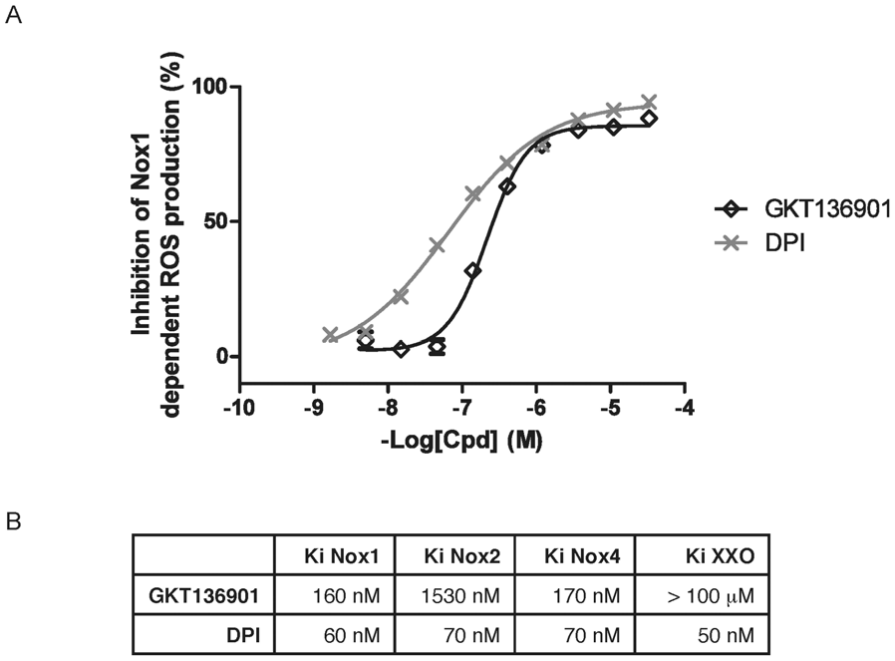
Inhibition of NOX1-dependent ROS production by GKT136901. (a) Concentration-response curve of GKT136901 (◊) and DPI (x) on membranes prepared from NOX1 over-expressing cells. Results represent one out of four experiments performed in triplicate. Values are presented as means ± s.e.m. (b) Affinities of GKT136901 and DPI on NOX1, NOX2, NOX4 and Xanthine Oxidase. GKT136901 is a NADPH-oxidase specific inhibitor, with selectivity on NOX1 and NOX4 over NOX2, whereas DPI inhibits all the NADPH oxidases tested with the same potency.

Next, we analyzed ROS production in murine and human endothelial cells in response to VEGF and bFGF stimulation. As expected, these factors increased intracellular ROS levels (Figure 2g–i), while in NOX1-deficient MLEC, ROS production in response to VEGF or bFGF stimulation was absent or severely compromised (Figure 2d,g). This is in contrast to NOX4 deficiency, which did not affect ROS production, induced by VEGF or bFGF. These data were consistent with observed deficient ROS production by endothelial cells following NOX1 silencing (Figure 2i and data not shown). Additionally, cells treated with the pharmacological NOX inhibitor GKT136901 did not induce ROS after VEGF or bFGF stimulation (Figure 2d–i). The specific NADPH oxidase inhibitor GKT136901 was identified by screening more than 130,000 molecules [28]. In a NOX subunit specific cell-free system [29], [30], GKT136901 inhibits NOX1 with high affinity (Ki  = 160±10 nM), similar to the irreversible flavoprotein inhibitor Diphenyliodonium (DPI; Ki  = 70±10 nM) (Figure 3a). However, DPI shows the same potency on NOX4 (Ki = 70 nM), NOX2 (Ki = 70 nM) and xanthine oxidase (Ki = 50 nM). In contrast, GKT136901 is more specific for NOX1 and NOX4 with a ten-fold lower potency on NOX2 (Ki  = 1530±90 nM) and almost no affinity for xanthine oxidase (Ki>100 µM) (Figure 3b and Figure S2). Moreover, GKT136901 completely inhibits oxidase activity of NOX1 and NOX4 but has only a partial effect on NOX2 (Figure S2). In order to demonstrate the specificity of GKT136901 for NOX enzymes, a pharmacological profiling of 135 different targets was performed including ROS producing and redox-sensitive enzymes [31]. GKT136901 at 10 µM showed low or no inhibition demonstrating the high degree of specificity of this compound (Table S1).

Taken together, the results obtained with NOX1 deficient cells and the inhibitor GKT136901 demonstrate that NOX1 is responsible for ROS production in endothelial cells stimulated with the pro-angiogenic factors VEGF or bFGF.

### NOX1 mediates endothelial cell migration and sprouting

**Figure 4:**
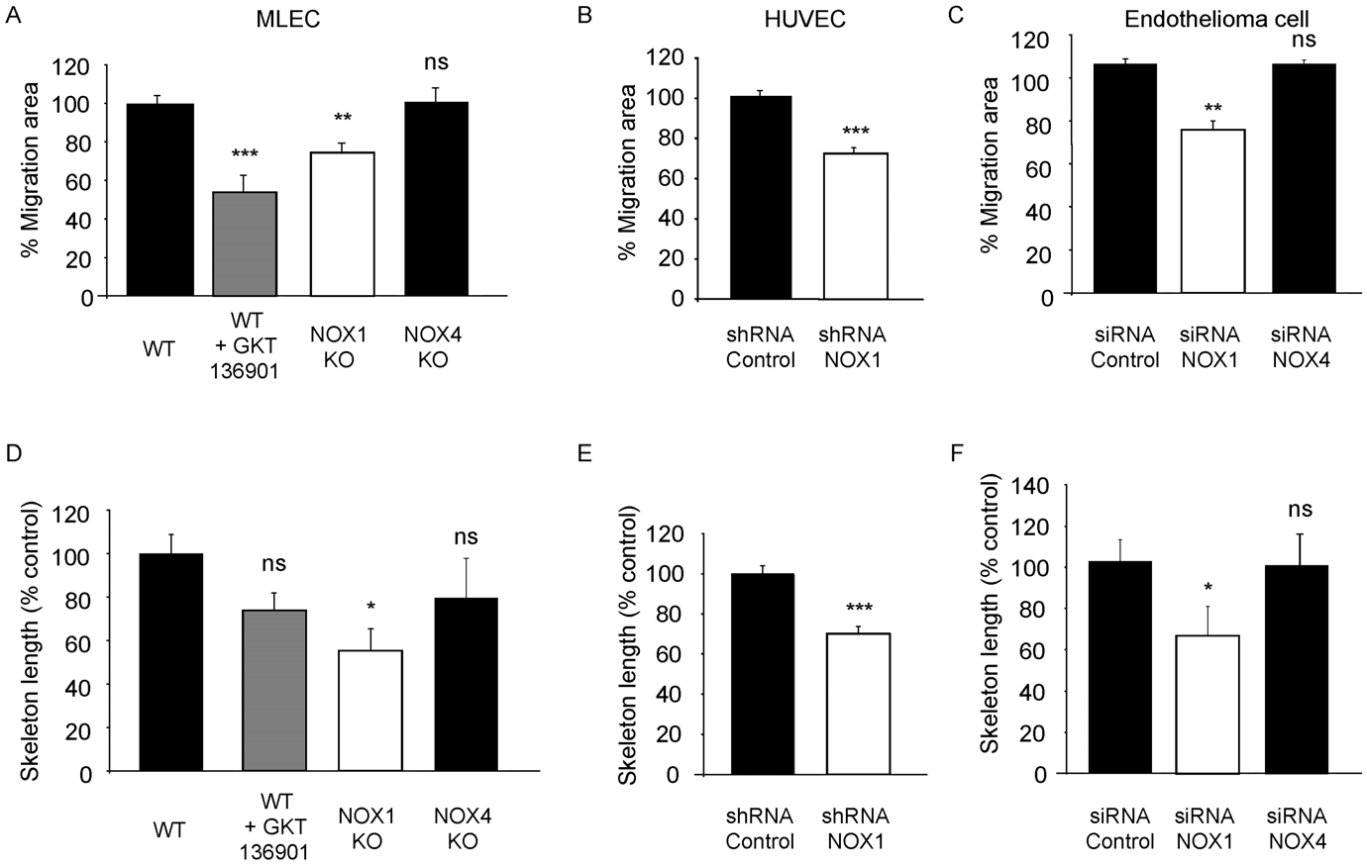
NOX1 inhibition blocks endothelial cell migration and tube-like structure formation. In vitro migration was analyzed by wound-healing assay on mouse primary endothelial cells (a), human primary endothelial cells (b) and endothelioma cell lines (c). Tubular structure formation was measured by 3D culture of mouse primary endothelial cells (d), human primary endothelial cells (e) or endothelioma cell lines (f). Results are expressed in % of control ± s.e.m, n = 3. *p<0.05, **p<0.01, ***<0.001 using Student's t-test. GKT136901 was used at 10 µM.

Angiogenesis requires endothelial cell proliferation, sprouting and migration [5]. To identify the NOX1-dependent step in angiogenesis, we performed different *in vitro* functional assays. Cell proliferation was not altered in NOX1-deficient MLEC, nor in NOX1 silenced cells (data not shown). However, cell migration and tube formation of MLEC from NOX1-deficient animals was significantly reduced as compared to WT cells (Figure 4a, d). We also observed an inhibition of endothelial cell migration and tube formation in MLEC treated with the NOX1 inhibitor GKT136901 (Figure 4) and NOX-dependent ROS blocking agents (Figure S3) [32], [33]. Furthermore, silencing of NOX1 expression using siRNA in mouse and human endothelial cells significantly reduced migration (17% ±5.7 and 24% ±3.8 of migrating area reduction respectively) and formation of tube-like structures (28% ±4.8 and 34% ±14 of reduction in skeleton length respectively) (Figure 4c, e and f). We did not observe any inhibition of migration or tube formation with MLEC derived from NOX4-deficient mice or with cells in which NOX4 was silenced using siRNA (Figure 4a, c, d and f).

Moreover, using a NOX1 expressing vector we observed that NOX1 overexpression (thirteen-fold increase) is sufficient to increase endothelioma cell migration and tube-like structure formation (Figure S4).

These experiments demonstrate that NOX1 is an important protein involved in migration and tube-like structure formation of endothelial cells with no detectable role in cell proliferation.

### NOX1 down-regulates PPARα expression

**Figure 5:**
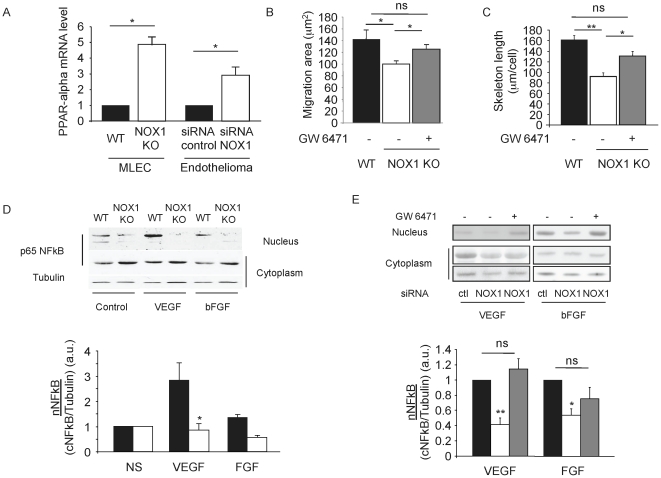
NOX1 negatively regulates PPARα expression and activity. NOX1 deficiency induces an up-regulation of PPAR**α** expression and inhibition of pro-angiogenic signaling in endothelial cells. (a). Expression level of PPAR**α** in MLEC and endothelioma cell lines by quantitative real-time PCR, normalized to the tubulin expression. (b–c) PPAR**α** antagonist, GW6471 compensated NOX1 deficiency in MLEC. (b). Migration of NOX1-deficient MLEC in presence or absence of PPAR**α** antagonist (10 µM). (c). Tube-like structure formation of NOX1-deficient MLEC in presence or absence of PPAR**α** antagonist (10 µM). VEGF and b-FGF induced-NFκB nuclear translocation is inhibited in absence of NOX1 and is dependent on PPAR**α** activity. (d). Western blot analysis of p65 NFκB in nuclear and cytoplasmic fractions of WT and NOX1-deficient cells stimulated 30 min with VEGF and bFGF (20 ng/ml). (e). Western blot analysis of p65 NFkB in nuclear and cytoplasmic fractions of NOX1-silenced cells treated or not with 10 µM of GW6471 before stimulation with VEGF and bFGF. The graphs show the abundance of nuclear p65 NF-κB relative to cytoplasmic p65 NF-κB ± s.e.m as determined by densitometry. n = 3. * p<0.05, ** p<0.01 (Student's t-test).

PPARα is a nuclear hormone receptor with anti-inflammatory functions able to block the angiogenic activity of VEGF [23], [34], [35]. We observed that NOX1-deficient MLEC expressed 5-fold higher PPARα compared to WT MLEC (Figure 5a), while no difference was seen with PPARγ expression (data not shown). This up-regulation of PPARα expression was also observed in endothelial cell lines silenced for NOX1 (Figure 5a), and was dependent on PPARα transactivation. Indeed, when NOX1-silenced cells were incubated with the PPARα antagonist GW6471, PPARα up regulation was no longer observed (data not shown). This suggests that the deficiency in migration and tube formation observed in NOX1-deficient endothelial cells may depend on up-regulation of PPARα expression and transcriptional activity.

To test this hypothesis, we treated NOX1-deficient MLEC with the PPARα antagonist GW6471 and found that the compound restored cell migration and tube formation compared to untreated NOX1-deficient cells (Figure 5b–c). Moreover, PPARα-deficient endothelial cells isolated from PPARα-deficient mice migrated more than WT endothelial cells. As expected, inhibition of NOX1 using the inhibitor GKT136901 in PPARα-deficient cells did not affect endothelial cell migration or invasion (data not shown).

Taken together, these results indicate that NOX1 promotes endothelial cell migration and sprouting by suppressing PPARα expression and activity.

### NOX1 promotes NF-κB activation in response to angiogenic stimulation by PPARα inhibition

We then set out to further identify the vascular NOX1 signaling pathways implicated in angiogenesis. Activation of both Akt and ERK1/2 were observed following 10 minutes of stimulation with the angiogenic factor bFGF. In NOX1-deficient MLEC, Akt activation was reduced compared to WT cells, while ERK1/2 activation was unaffected (Figure S5). These results showed that NOX1 is involved in the activation of the Akt signaling pathway in response to bFGF. Since NOX1 suppresses PPARα expression, this is also consistent with the literature describing PPARα as inhibitor of VEGF signaling through blocking of Akt but not ERK1/2 activation.

Since PPARα is known to inhibit NF-κB activation, we monitored NF-κB activation in NOX1-deficient MLEC in response to VEGF or bFGF stimulation (30 minutes). NOX1-deficient MLEC showed reduced nuclear translocation of the NF-κB p65 subunit compared to WT cells in response to VEGF or bFGF as demonstrated by Western blotting on nuclear extracts and immunofluorescence of endothelial cells (Figure 5d and Figure S6). Using endothelial cell lines, we then demonstrated that inhibition of NF-κB translocation depended on PPARα activation. Indeed, in NOX1-silenced cells PPARα expression was up-regulated (Figure 5a). When these cells were treated with the PPARα antagonist GW6471 before angiogenic stimulation, deficient nuclear translocation of NF-kB was no longer observed (Figure 5e and Figure S6). Moreover in NOX1 deficient cells, PPARα and NF-κB target genes were deregulated (Table 1). For example: Vascular NOX1 deficiency leads to upregulation of the anti-oxidant genes catalase, gluthatione peroxydase-3 and the anti-migratory gene VE-Cadherin, while downregulating the proangiogenic genes MMP2, MMP9, uPAR, VEGF and bFGF. Incubation of NOX1 silenced cells with the PPARα antagonist GW6471 blocked this effect, suggesting that regulation of these was dependent on PPARα activity (Table 1).

**Table 1:** Expression of genes regulated by NOX1 deficiency.

	LMEC NOX1 KO	tEnd siRNA NOX1	tEnd siRNA NOX1 + GW 6471
Catalase	**1.52±0.29**	**1.52±0.19**	**0.92±0.012**
GPX3	**1.47±0.08**	**1.49±0.13**	**0.70±0.01**
VE-cadherin	**2.29±0.07**	**1.54±0.28**	**0.9±0.2**
MMP-2	**0.43±0.24**	**0.97±0.10**	**2.13±0.24**
MMP-9	**0.29±0.13**	**0.89±0.13**	**0.47±0.07**
uPAR	**0.41±0.12**	**0.76±0.19**	**1.34±0.6**
VEGF	**0.31±0.05**	**2.04±0.29**	**0.68±0.04**
bFGF	**0.71±0.21**	**0.81±0.13**	**0.64±0.25**
VCAM-1	**0.84±0.01**	**0.76±0.04**	**0.57±0.18**

Level of expression of target genes in NOX1 deficient primary endothelial cells (MLEC), or silenced cells endothelial cell lines compared to control cells, analyzed by quantitative real-time PCR. Results are expressed in fold increase or decrease ± s.e.m. n = 3.

### Host NOX1 promotes tumor angiogenesis and tumor progression

**Figure 6:**
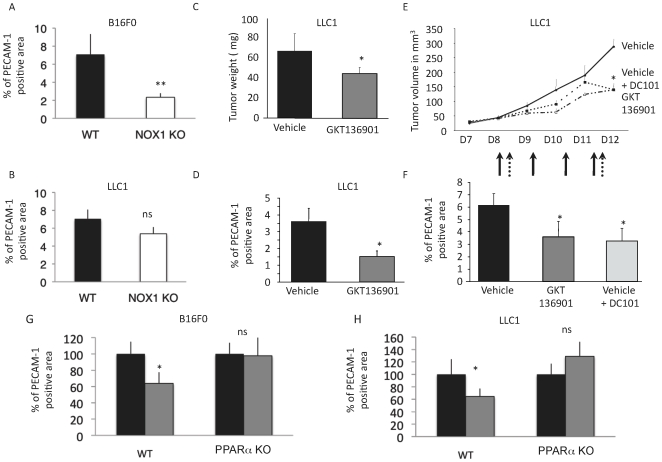
NOX1 inhibition reduces tumor growth and angiogenesis. B16F0 (a) or LLC1 (b) tumor cells were injected subcutaneously into WT or NOX1 KO mice. Tumors were allowed to develop for 10 days and the density of tumor vasculature was identified using PECAM-1 staining by measuring the positive (vascular) and total (tumor) area ± s.e.m expressed as percentage. n = 8; WT mice injected with LLC1 tumor cells were then treated with the NOX inhibitor GKT136901 at 40 mg/kg/day by oral administration for 8 days; (c). Graph represents tumor weight in mg ± s.e.m n = 8 per group. (d). Tumor vessel density in tumors from the experiment in (c). Graph shows tumor vascularization expressed in percentage of PECAM-1 positive area ± s.e.m.; (e). Changes in tumor volume in mm^3^ after therapeutic treatment starting 8 days post tumor cell injection with GKT136901 (black arrows) or anti-VEGFR2 (DC101) (pointed arrows). Tumor volume is measured using a caliper and the formula V = 4/3π(L/2*l/2*w/2). n = 8; (f). Blood vessel density in tumors from the experiment in (e). at day 12. Graph shows tumor vascularization expressed in percentage of PECAM-1 positive area ± s.e.m. WT or PPAR**α** KO mice injected with B16F0 (g) or LLC1 (h) were then treated with the NOX inhibitor GKT136901 at 40 mg/kg/day by oral administration for 8 days. Graph shows tumor vascularization expressed in percentage of PECAM-1 positive area ± s.e.m. n = 6. * p<0.05, ** p<0.01 (Student's t-test).

The above findings led us to investigate whether NOX1 may contribute to tumor progression by promoting tumor vascularization. To this end, we implanted tumorigenic B16F0 melanoma cells or Lewis Lung Carcinoma (LLC1) cells subcutaneously into WT and NOX1-deficient mice. LLC1 cells expressed high level of NOX1 in contrast to B16F0 cells. We observed reduced tumor vascularization in NOX1-deficient animals with B16F0 melanoma but not with LLC1 tumors (Figure 6a–b).

To assess whether NOX1 may be a valuable target for cancer therapy, we used the inhibitor GKT136901. This inhibitor did not interfere with tumor cell proliferation and apoptosis *in vitro* but it impinged in their ROS producing activity (Figure S7). One day after LLC1 injection, tumor bearing mice were treated daily by oral administration of GKT136901 at 40 mg/kg. After one week of treatment, the size of tumor in GKT136901-treated mice was 34% ±5.8 smaller compared to tumors in vehicle-treated mice (Figure 6c). This treatment had no toxic effect on mice as illustrated by the Figure S8. Quantification of tumor vasculature by PECAM-1 immunostaining showed 59% ±9.3 reduction of vascular area in GKT136901-treated tumors as compared to vehicle treated animals (Figure 6d). In addition, we performed a therapeutic assay by treating animals bearing established tumors (i.e. starting 8 days after tumor implantation) with GKT136901 or the anti-VEGFR2 antibody DC101, as a positive control. Tumor volume was measured daily during treatment. Both treatments delayed tumor progression (35% ±5.7 and 35% ±9.7 respectively) and vascularization (36% ±16 and 43% ±20 respectively) (Figure 6e, f). From these results, we conclude that NOX1 plays a critical role in promoting tumor angiogenesis and tumor progression.

To further analyze the link between NOX1 and PPARα *in vivo*, we injected LLC1 or B16F0 tumor cells in either WT or PPARα-deficient mice and treated these animals with the NOX1 inhibitor GKT136901. Vascularization of these tumors was analyzed by PECAM-1 immunostaining. In WT mice, the inhibitor blocked LLC1 and B16F0 tumor vascularization (100% ±24 vs 65% ±13 and 100% ±15 vs 64% ±14), whereas it had no effect in PPARα deficient mice (100% ±17 vs 129% ±23 and 100% ±26 vs 98% ±24) (Figure 6g,h).

From these observations, we conclude that NOX1 promotes tumor angiogenesis by inhibiting the anti-angiogenic factor PPARα.

## Discussion

**Figure 7:**
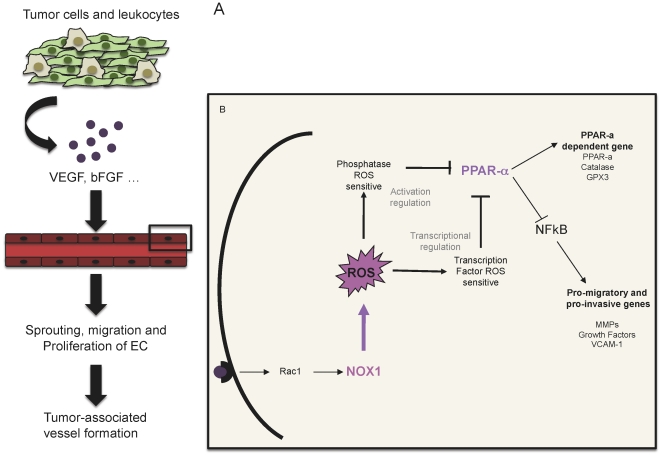
Schematic representation of the role of NOX1 in tumor angiogenesis. (a) Tumor cells and tumor-infiltrating leukocytes during tumor development produce proangiogenic factors such as VEGF and bFGF. These factors activate the preexisting vessels to form neovessels by sprouting, migration and proliferation. (b) Endothelial cells receive the angiogenic stimulus by fixation of the angiogenic factor to the surface receptor. This interaction initiates a signaling cascade, which leads to NOX1 activation through Rac1. NOX1 dependent ROS inhibits the nuclear hormone receptor PPAR**α** by post-translational modification and transcriptional regulation. This inhibition leads to NF-κB activation and transcription of angiogenic factors such as MMPs, growth factors or promigratory molecules.

Reactive oxygen species are important players in cancer biology [36]–[38]. At high levels, they induce apoptosis and/or senescence while at homeostatic levels they influence survival and proliferation. In tumor endothelial cells, NOX enzymes contribute to angiogenesis through ROS-dependent mechanisms, and thereby promote tumor growth. In this study, we show that vascular NOX1, but not NOX2 or NOX4, is an important regulator of angiogenesis, through the modulation of endothelial migration and tube formation. We furthermore identified that NOX1 activity suppresses the anti-inflammatory nuclear hormone receptor PPARα as a critical mechanism mediating these effects (Figure 7).

Inhibition of NOX1 decreases endothelial cell migration. A link between NOX1, ROS and cell migration has been previously described with non-endothelial cells such as colon carcinoma cell lines [39]. In addition, Schröder et al. reported that blocking NOX1 reduced FGF-stimulated migration of smooth muscle cells through NOX1 dependent ROS activation of JNK, which in turn phosphorylated paxillin [40]. Modulating smooth muscle cell migration may not only be restricted to NOX1, as NOX4 showed similar effects [41]. However, these conflicting data may be due to the different mode of smooth muscle cell activation by bFGF and PDGF, respectively. Based on these data, it is clear that NOX1 and derived ROS products impede the migration of vascular smooth muscle and tumor cells. The role of NOX1 in endothelial cell migration and angiogenesis was previously neglected probably due to the fact that in resting endothelial cells NOX1 is expressed at low levels and is up-regulated only during the angiogenic switch as we show in this study.

Importantly, we do not find evidence for the involvement of the other tested NOX isoforms 2 and 4 in bFGF-induced angiogenesis in vivo. This is in contrast to other reports suggesting an angiogenic role for these NOX isoforms. Angiopoietin-1, VEGF, or thrombin-induced neovascularization in vitro and in vivo have been suggested to be dependent on NOX2 [42]–[45]. Furthermore, a recent study showed that inhibitors specific for NOX4 and NOX2 blocked hemangioma (i.e. endothelial cell-derived tumor) growth in vivo [33]. Other laboratories found NOX4 responsible for basal endothelial ROS production and cell proliferation [46]. The function of NOX2 and NOX4 in angiogenesis may depend on the type of stimulation and operate by a different mechanism than NOX1, as both enzymes are involved in endothelial cell proliferation, while NOX1 mediates endothelial cell migration and sprouting but not proliferation.

Our results reveal a remarkable specificity of NOX isoforms. Clearly, endothelial cells express NOX1, NOX2, NOX4, and – at least in humans - also NOX5. Yet, their functions appear to be non-redundant. The specificity of NOX isoforms relies on several elements, including different subcellular localization (NOX4 is predominantly intracellular, while NOX1 rather localizes to caveolae [47]), different activation mechanisms (NOX4 appears constitutively active, while NOX1 and NOX2 are activation-dependent, [10], [48]), and there is also isoform-specific upregulation of NOX mRNA in response to given stimuli. Most likely three elements contribute to the unique role of NOX1 in angiogenesis: i) its localization within caveolae is in proximity of other signaling molecules, ii) to mediate VEGF signaling, it should be an activation inducible enzyme, rather than constitutively active such as NOX4; and iii) as shown in Fig. 2, its mRNA is indeed up-regulated in response to VEGF, which adds specificity to the system. Thus, the understanding of which NOX isoforms are relevant for angiogenesis, provided by our study adds new understanding of redox biology. However, the precise identification of the NOX isoform involved in a given pathophysiological process will also be the key for the development of specifically targeted NOX inhibitors [49].

With respect to tumor growth, we observed differences between NOX1-deficient mice and wildtype mice treated with the NOX inhibitor. Indeed, while plug- and tumor-induced angiogenesis was efficiently decreased in the NOX1-deficient mouse and by the NOX inhibitor, the latter was markedly more efficient in decreasing tumor growth. The most likely explanation for this difference is the possibility that NOX enzymes within the tumor cells are contributing to tumor growth and that the NOX inhibitor targets these enzymes. Indeed, there is ample evidence in the literature for a role of NOX enzymes in enhancing growth of tumor cells [50]–[53]. Moreover, we found that vascularization was substantially diminished in B16 tumors lacking endogenous NOX1 expression, while NOX1-expressing LLC1 tumors were normally vascularized in NOX1 deficient mice. This apparent discrepancy is consistent with the literature, showing that exogenous ROS impacts on endothelial cell proliferation, migration and tube formation [54]–[56]. Induction of angiogenesis may be due to NOX1 expression by LLC1 tumors, producing high amounts of ROS in the tumor microenvironment and modulating endothelial cell functions. This amount of ROS seems to be sufficient to stimulate endothelial cell proliferation and migration to form new vessels within the tumor. In the GKT136901-treated mice, the tumor cells cannot stimulate ROS mediated angiogenesis as the inhibitor also blocks ROS production by LLC1 tumor cells (Figure S6c). Another obvious difference between the NOX1-deficient mice and the NOX inhibitor is the fact that GKT136901 efficiently inhibits NOX4, in addition to NOX1. This point is particularly pertinent because previous studies have implicated NOX4 in mechanisms of angiogenesis. To understand these apparent differences, angiogenic responses should be subdivided in a proximal response of the hypoxic tissue (HIF1α activation and VEGF production) and a distal response of the endothelial cells (VEGF-induced neovascularization). Our results (Fig. 1) clearly demonstrate that NOX4 is not involved in the distal response of endothelial cells. However, our results do not argue against a role of NOX4 in the proximal response of hypoxic tissues. Indeed, based on the literature [57], [58], we think that such a role of NOX4 is likely.

Under homeostatic conditions, ROS levels are balanced by scavenger and antioxidant enzymes. As in certain pathologies this balance becomes deregulated and much effort has been put into development of inhibitors of ROS production [59]. Since most ROS-related diseases are mediated by one NOX isoform only, novel inhibitors, which are isoform specific, will be invaluable. The novel GenKyoTex inhibitor, GKT136901, has been developed for this purpose and appears to be specific for NOX1 and NOX4 with similar affinities. In vitro, this inhibitor blocked ROS production induced by proangiogenic factors and inhibited endothelial cell migration. More importantly, the level of this inhibition was comparable to that observed in NOX1-deficient cells. With NOX4-deficient endothelial cells however, we observed no reduced migration. From these data we conclude that endothelial cell migration is NOX1 dependent and NOX4 independent. Furthermore, the potential for GKT136901 to be used as a NOX1/4 specific inhibitor in prevention of tumor angiogenesis is also demonstrated.

We observed an increase of PPARα expression when NOX1 was not expressed; suggesting that NOX1 activity constitutively represses PPARα expression. As previously mentioned, in the absence of NOX1 expression, endothelial cells were less able to migrate and to form tubular structures, we showed that this effect was reversed using an antagonist of PPARα. Moreover, in NOX1-deficient endothelial cells, NFκB was not activated after VEGF or bFGF stimulation. This effect was reversed by treatment with a PPARα antagonist. In addition, anti-oxidants and anti-migratory genes were up-regulated and pro-angiogenic genes down-regulated by NOX-1 deficiency. These differences in gene expression depended on PPARα transactivation and they explain the reduced migratory phenotype and tube formation of NOX1 deficient endothelial cells. PPARα activity, which itself controls PPARα expression, could be directly regulated by the catalytic activity of NOX1. Indeed, PPARα activity is regulated by several post-transcriptional modifications such as phosphorylation or SUMOylation [60], [61], which may be ROS sensitive [62]. It has been previously shown that ROS inactivates phosphatases [63], [64] and activates SUMOylation [62], [65]. Thus, upregulation of PPARα expression and activity in NOX1-deficient cells blocks angiogenic signaling needed for endothelial cell migration, sprouting and angiogenesis.

Currently, the anti-VEGF antibody bevacizumab (Avastin), and several small molecular VEGFR tyrosine kinase inhibitors, are used as anti-angiogenic drugs to treat patients with advanced cancers [66], [67]. Anti-angiogenic therapy targets non-transformed endothelial cells and results in the reduced delivery of nutrients and oxygen to tumor cells. However, concomitant chemotherapy is needed in order to obtain a survival advantage. Emerging evidence indicates that tumors treated with anti-angiogenic therapy elicit compensatory reactions by inducing the production of alternative angiogenic factors. Thus, the question arises as to whether anti-NOX therapy could potentially be a therapeutic approach complementary to anti-VEGF treatment. Indeed, expression of NOX molecules are induced in vascular endothelium and in tumor cells during the angiogenic switch and inhibition of NOX1 in both the host and the tumor cells using the potent NOX1 inhibitor GKT136901, results in reduced tumor angiogenesis and tumor growth. Moreover NOX1 seems to form a common angiogenic pathway, at least for signaling induced by VEGF and FGF. As a result, NOX1 inhibition could bypass the potential compensatory mechanisms activated by anti-VEGF therapy.

In conclusion, we have identified NOX1 as a novel mediator of angiogenesis and as a candidate therapeutic target for anti-angiogenic therapies in cancer.

## Materials and Methods

### Mice

NOX1, NOX2, NOX1/2, NOX4 deficient mice were inbred on the C57BL/6J background for more than 6 generations. The PPARα null animals were originally described in [68]. All animal procedures were performed in accordance with the Institutional Ethical Committee of Animal Care in Geneva and Cantonal Veterinary Office. The Institutional Ethical Committee of Animal Care in Geneva and Cantonal Veterinary Office specifically approved this study through experimentation IDs: 31.1.1005-3456-0, 1005-3325-1, 1005-3329-2.

### Cells and reagents

Endothelial cell line (thymus derived endothelioma [69]) was cultured in Dulbecco Modified Eagle medium supplemented with 10% FCS, 100 U/ml Penicillin/Streptomycin, 100 U/ml Glutamine. Primary lung endothelial cells were cultured in DMEM/HAM F-12 medium supplemented with 20% of FCS, 100 U/ml Penicillin/Streptomycin, 100 U/ml Glutamine, 100 µg/ml Heparin (Sigma-Aldrich), 10 µg/ml Endothelial cell growth supplement (Upstate). MLEC were used from passage 4 to 6. Human umbilical vein endothelial cells (HUVEC) were isolated in the laboratory, cultured in Bullet kit (Lonza) and used from passage 4 to 6. Gelatin, Fibronectin, Fibrinogen, and Aprotinin were obtained from Sigma Aldrich. Growth factor reduced Matrigel was from Becton Dickinson. Murine and human bFGF, human and murine VEGF were from Peprotech. Antibodies against phospho-p42/44 MAPKinase, total MAPKinase, phosphoThr308 Akt and total Akt were purchased from Cell Signaling Technology. Antibody against NF-kB p65 was purchased from SantaCruz Biotechnology and anti-Actin antibody was kindly provided by Dr Christine Chaponnier. DC101 hydridoma was purchased from ATCC (Manassas, VA) and DC101 antibody was expressed and purified at the protein expression facility at EPFL, Lausanne.

### In vivo angiogenesis assay

7 to 10 week old females were injected subcutaneously with 400 ml of growth factor reduced Matrigel supplemented with 500 ng/ml of bFGF. One week later, mice were scanned using Micro-CT (Skyscan-1076). Mice were scanned before and after retro-orbital injection of 400 ml iodinated liposomes (BR22, BracoResearch, Plan-les-Ouates) to visualize the vessel density in the plug as described previously [70], [71].

### MLEC isolation

Murine lung endothelial cells were isolated as described previously [27]. Briefly, whole mouse lungs were digested in collagenase type I 0.1% (Gibco). Digest lung were plated on gelatin/collagen/fibronectin-coated flask in DMEM/HAM F-12 medium supplemented with 20% of FCS, 100 U/ml Penicillin/Streptomycin, 100 U/ml Glutamine, 100 µg/ml Heparin (Sigma-Aldrich), 10 µg/ml Endothelial cell growth supplement (Upstate). The following day, macrophages were depleted from the culture by negative selection using a rat anti-mouse FcgRII/III antibody coupled to anti-rat coated magnetic beads (Dynal). Cells were then positively selected twice using the endothelial marker, PECAM-1 (Figure S3).

### Quantitative RT-PCR

Total RNA from treated cells was extracted using RNeasy minikit (Qiagen). Total RNA was reverse-transcribed with the Superscript III first strand RT-PCR kit (Invitrogen). Quantitative real-time PCR was performed using SybrGreen master mix (Applied Biosystems) on Step one plus Real-time PCR machine (Applied Biosystems). Primer sequences are listed in the Materials and Methods S1.

### Detection of superoxide

Endothelial cells were seeded on glass slides and stained with dihydroethidium (DHE). Images were captured with an inverted microscope and analyzed with Metafluor imaging software (MDS Analytical Technologies). Quantification was performed by measuring the fluorescence intensity of over minimum 50 endothelial cells per slide.

### NOX inhibition in cell free assays and pharmacological profile of GKT136901

Membranes from NOX2 expressing PMN cells or from cells overexpressing NOX1 or NOX4 were prepared as previously described [29]. After resuspension in sonication buffer (11% sucrose, 120 mM NaCl, 1 mM EGTA in PBS, pH 7.4 for NOX4-expressing cells) or in relax buffer (10 mM Pipes, 3 mM NaCl, 3.5 mM MgCl2, 0.1 M KCl, pH 7.4), cells were broken by sonication and centrifuged (200 g, 10 min). The supernatant was loaded onto a 17/40% (w/v) discontinuous sucrose gradient and centrifuged (150,000 g for 30 min). Membrane fractions were collected and stored at −80°C. ROS production measurements of membranes expressing different NOX subunits was determined as previously described [30] using the Amplex Red method (Invitrogen). Membranes prepared from non-transfected cells did not show NADPH-induced ROS production (data not shown).

### Gene silencing

For mouse cells, siRNAs were nucleofected using the Amaxa technology (Lonza). Gene silencing was assessed 48 hours after nucleofection by quantitative RT-PCR. For HUVEC, shRNA vectors (SABiosciences) were nucleofected using Amaxa. Gene silencing was assessed 48 hours after nucleofection by quantitative RT-PCR.

### Wound healing assay

Wound healing assay was performed as described previously [72]. Briefly, endothelial cells were plated in Matrigel (Becton Dickinson) precoated 96 wells plates. One day after plating the cell monolayer was scratched to make a regular wound. Cells were allowed to migrate overnight. Migration area was then measured and calculated using the Metamorph program (MDS Analytical Technologies).

### Endothelial cell sprouting assay

Sprouting assay was performed as described previously [72]. Briefly, endothelial cells were plated in 96 well plates at 8,000 cells per well in a 3D fibrin gel. Above the gel, 10% FCS containing DMEM was complemented with 200 KIU/ml of Aprotinin (Sigma). Length of branches was evaluated using the Metamorph program. Results are expressed in mm of skeleton length/number of cells.

### Nuclear and cytoplasmic extraction

After stimulation, nuclear and cytoplasmic proteins of endothelial cells were extracted according to Tauzin et al. [73]. Briefly, cells were incubated in TKM buffer (50 mM Tris-HCl pH 7.4, 25 mM KCl, 5 mM MgCl_2_ and 1 mM EGTA) containing 1% Triton and protease inhibitor cocktail CLAP [10 µg/ml chymostatin, leupeptin, antipain and pepstatin A (Sigma) for 30 minutes on ice, sonicated for 2 minutes and centrifuged at 5000 g for 30 minutes. The pellet and the supernatant were separated and solubilized in sample buffer.

### Western blotting

Cells were stimulated for the indicated time and then lysed in TNT buffer (50 mM Tris, 150 mM NaCl, 0.5% Triton X-100) complemented with protease inhibitor cocktail CLAP and phosphatase inhibitor cocktail [25 mM NaF, 20 mM b-Glycerophosphate, 5 mM HEPES, 2.5 mM EDTA, 0.5 mM Orthovanadate]. Membranes were blocked in PBS containing 0.5% BSA and hybridized with different antibodies. Blots were revealed with peroxidase coupled secondary reagent (Jackson Immunoresearch) followed by ECL and quantified by densitometry using Image J software.

### Tumor growth assay

5. 10^5^ of LLC1 or B16F0 were injected subcutaneously on the back of mice. Mice were treated with NOX inhibitor, GKT136901 or vehicle (Carboxymethyl-cellulose, CMC) at 40 mg/kg everyday per os or i.p with anti-VEGFR2 antibody (DC101) at 0.8 mg every 2 days. When the control tumor reached approximately 1 cm in length, mice were sacrificed and the tumor excised, weighed and frozen. Frozen sections of tumors were stained with anti-PECAM-1 antibody (rat monoclonal, [74]).

### Statistical analysis

All statistical analysis was performed using Anova on multiple variable analyses and Student's t-test on paired analyses. *(p = 0,05), **(p = 0,01), ***(p = 0,001).

## Supporting Information

Table S1 Inhibitory effect of the inhibitor GKT 136901 on ROS producing enzymes, redox-sensitive enzymes and others proteins.
(0.03 MB DOC)

Figure S1 MLEC isolation from WT and NOX deficient mice. a. Flow cytometry analysis of endothelial surface molecules on isolated MLEC. PECAM-1, VE-Cadherin, ICAM-2 and Meca-32 expression level in MLEC. b. PECAM-1 immunofluorescence staining of WT, NOX1 KO and NOX4 KO MLEC. Nuclei in blue (DAPI), and PECAM-1 in purple (Cy5). Images were acquired with a 40x/1.3 numeric aperture lens and analyzed using LSM510 Meta microscope (Carl Zeiss).
(6.76 MB TIF)

Figure S2 Inhibition of NOX-dependent ROS production by GKT136901 and DPI. a. Concentration-response curves of GKT136901 on NOX1 (x), NOX2 ▴), NOX4 (◊) and Xanthine Oxidase (XO) (□). b. Concentration-response curve of DPI on NOX1 (x), NOX2 (▴), NOX4 (◊) and Xanthine Oxidase (XO) (□) Results are from one experiment performed in triplicate, representative of four performed. Values are presented as means ± s.e.m.
(2.02 MB TIF)

Figure S3 NOX dependant ROS blocking agents efficiently block endothelial cell migration and branching capacities. a. Migration of endothelial cells was analyzed by a wound-healing assay in presence of different inhibitors that block NADPH dependant ROS production. b. Tubular structure formation was measured by 3D culture using the mouse endothelial cell line in presence of different inhibitors that block NADPH dependant ROS production. Results are expressed in % of control ± s.e.m, n = 3.
(5.89 MB TIF)

Figure S4 NOX1 over-expression enhances endothelial cell migration and tube-like structure formation. a. In vitro migration was analyzed by wound-healing assay using endothelioma cell lines transfected with NOX1 expressing vector. b. Tubular structure formation was measured by 3D culture of endothelioma cell lines transfected with NOX1 expressing vector. Results are expressed in % of control ± s.e.m. *p< 0.05 using Student's t-test.
(5.88 MB TIF)

Figure S5 AKT but ERK 1/2 activation is affected by NOX1 deficiency. NOX1-deficient MLEC does not activate Akt after bFGF stimulation but present no difference in ERK1/2 activation. a. Western blot analysis of Akt phosphorylation in WT and NOX1-deficient MLEC after 10 min stimulation with 20 ng/ml of bFGF. The graph shows the abundance of phosphorylated Akt relative to total Akt ± s.e.m as determined by densitometry. n = 3. b. Western blot analysis of ERK1/2 phosphorylation in WT and NOX1-deficient MLEC stimulated for 10 min with 20 ng/ml of bFGF. The graph shows the abundance of phosphorylated ERK1/2 relative to total ERK1/2 ± s.e.m as determined by densitometry. n = 3.
(6.13 MB TIF)

Figure S6 NF-κB nuclear translocation is inhibited in the absence of NOX1 and dependent on PPARα activation. VEGF or b-FGF stimulation of endothelial cells induced p65 NF-κB translocation into the nucleus. This nuclear translocation is not observed in NOX1-deficient cells but restored by PPARα antagonist treatment (GW6471). Immunofluorescence, anti-p65 NF-κB of MLEC (a) and endothelioma cell lines (b) stimulated with VEGF or bFGF in presence or absence of GW6471 (10mM). NF-κB in green (Alexa 488), nuclei in blue (DAPI). Scale bar represent 20 mm. Images were acquired with a 40x/1.3 numeric aperture and analyzed using LSM510 confocal microscope (Carl Zeiss).
(7.09 MB TIF)

Figure S7 Effect of GKT 136901 on tumor cells. a. LLC1 and B16F0 cell proliferation was measured by EdU incorporation and propidium iodide staining of DNA content, 24h after incubation with 10 μM of GKT136901. b. LLC1 apoptosis was measured by AnnexinV/PI staining after 24h of incubation with 10 μM of GKT136901. c. ROS levels produced by LLC1 are inhibited by GKT136901. ROS production was quantified by DHE substrate 1h after incubation with 10 μM of the inhibitor. *** p<0.001 (student t-test).
(6.11 MB TIF)

Figure S8 Non toxic effect GKT 136901 on mice organs. Heart (a), Kidney (b), Liver (c) and Lung (d) of mice treated orally with vehicle or with vehicle plus GKT136901 inhibitor at 40 mg/kg per day during 8 days, stained by Hematoxilin/Eosin. Scale bars represent 100 μm on the full picture and 20 μm on the zoom. Images were acquired with a 20x/0.8 numeric aperture and analyzed using Mirax (Carl Zeiss).
(8.38 MB TIF)

Materials and Methods S1 Real-time PCR primer sequence list.
(0.05 MB DOC)
