# Correction: Targeting Vascular NADPH Oxidase 1 Blocks Tumor Angiogenesis through a PPARα Mediated Mechanism

**DOI:** 10.1371/annotation/a392bbef-b0ec-4c70-b403-74a7bad85178

**Published:** 2011-02-17

**Authors:** Sarah Garrido-Urbani, Stephane Jemelin, Christine Deffert, Stéphanie Carnesecchi, Olivier Basset, Cédric Szyndralewiez, Freddy Heitz, Patrick Page, Xavier Montet, Liliane Michalik, Jack Arbiser, Curzio Rüegg, Karl Heinz Krause, Beat A. Imhof

The 14th author's name is incorrect. The correct name is: Beat A. Imhof 

