# Supplementary material for: Targeting Vascular NADPH Oxidase 1 Blocks Tumor Angiogenesis through a PPARα Mediated Mechanism
Source: PLoS One. 2011 Feb 7;6(2):e14665. doi: 10.1371/journal.pone.0014665 (PMC3034713; doi:10.1371/journal.pone.0014665)
Supplement: Table S1 — Inhibitory effect of the inhibitor GKT 136901 on ROS producing enzymes, redox-sensitive enzymes and others proteins. (0.03 MB DOC) [file pone.0014665.s001.doc]

| **Enzyme** | **% Inhibition at 10 μM** |
| --- | --- |
| Xanthine oxidase | 5% |
| iNoS | 0% |
| eNos | 6% |
| cNos | 0% |
| MPO | 0% |
| 12-Lipoxygenase | 2% |
| MAO-B | 0% |
| PKC a, bI, bII, d | 0% |
| PI3K a, b, d, g | 0% |

**Table S1.**

Inhibitory effect of the inhibitor GKT 136901 on ROS producing enzymes, redox-sensitive enzymes and others proteins.
