# Supplementary figures and images for: Targeting Vascular NADPH Oxidase 1 Blocks Tumor Angiogenesis through a PPARα Mediated Mechanism
Source: PLoS One. 2011 Feb 7;6(2):e14665. doi: 10.1371/journal.pone.0014665 (PMC3034713; doi:10.1371/journal.pone.0014665)

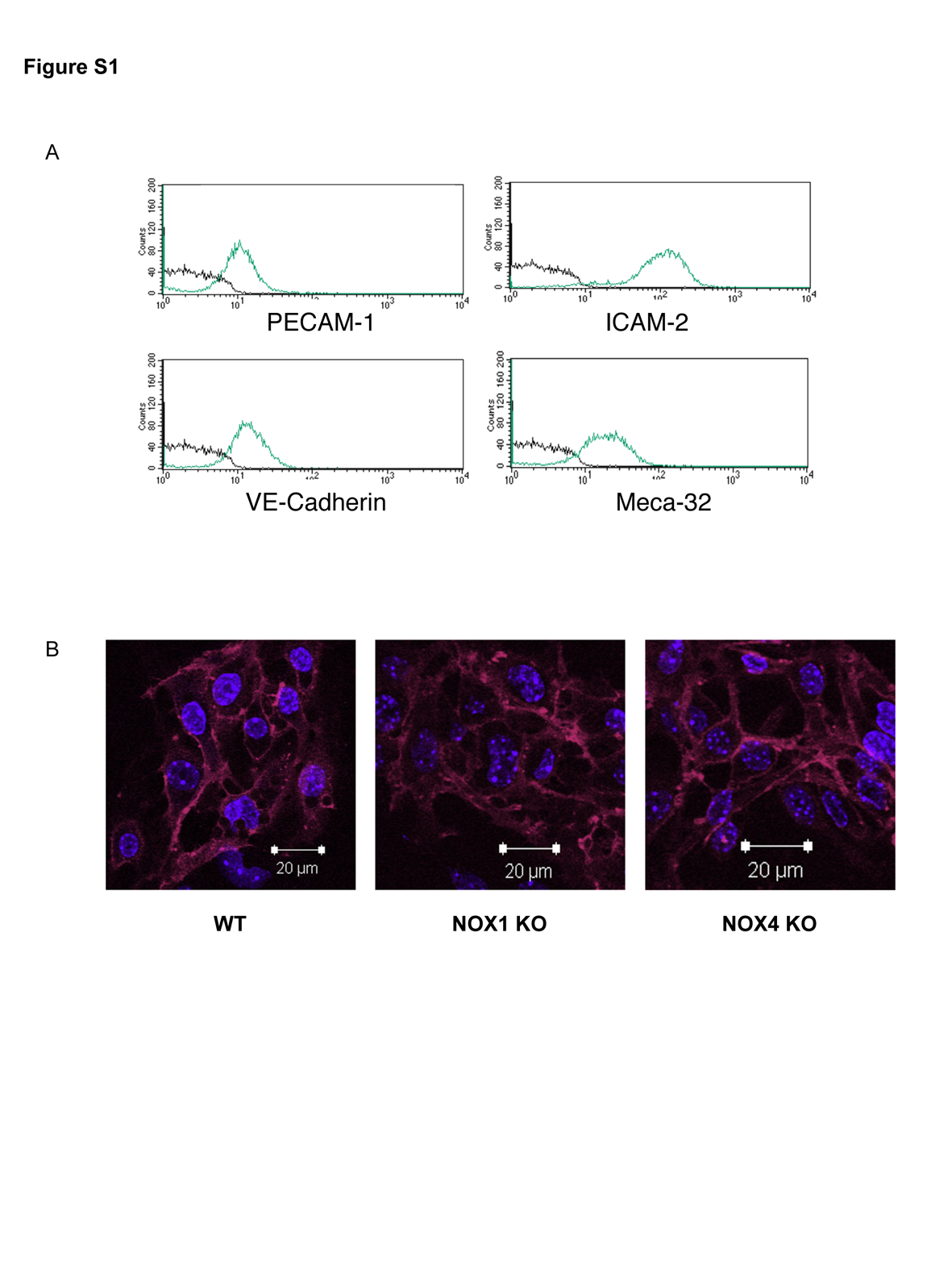

Supplement: Figure S1 — MLEC isolation from WT and NOX deficient mice. a. Flow cytometry analysis of endothelial surface molecules on isolated MLEC. PECAM-1, VE-Cadherin, ICAM-2 and Meca-32 expression level in MLEC. b. PECAM-1 immunofluorescence staining of WT, NOX1 KO and NOX4 KO MLEC. Nuclei in blue (DAPI), and PECAM-1 in purple (Cy5). Images were acquired with a 40x/1.3 numeric aperture lens and analyzed using LSM510 Meta microscope (Carl Zeiss). (6.76 MB TIF) [file pone.0014665.s002.tif]

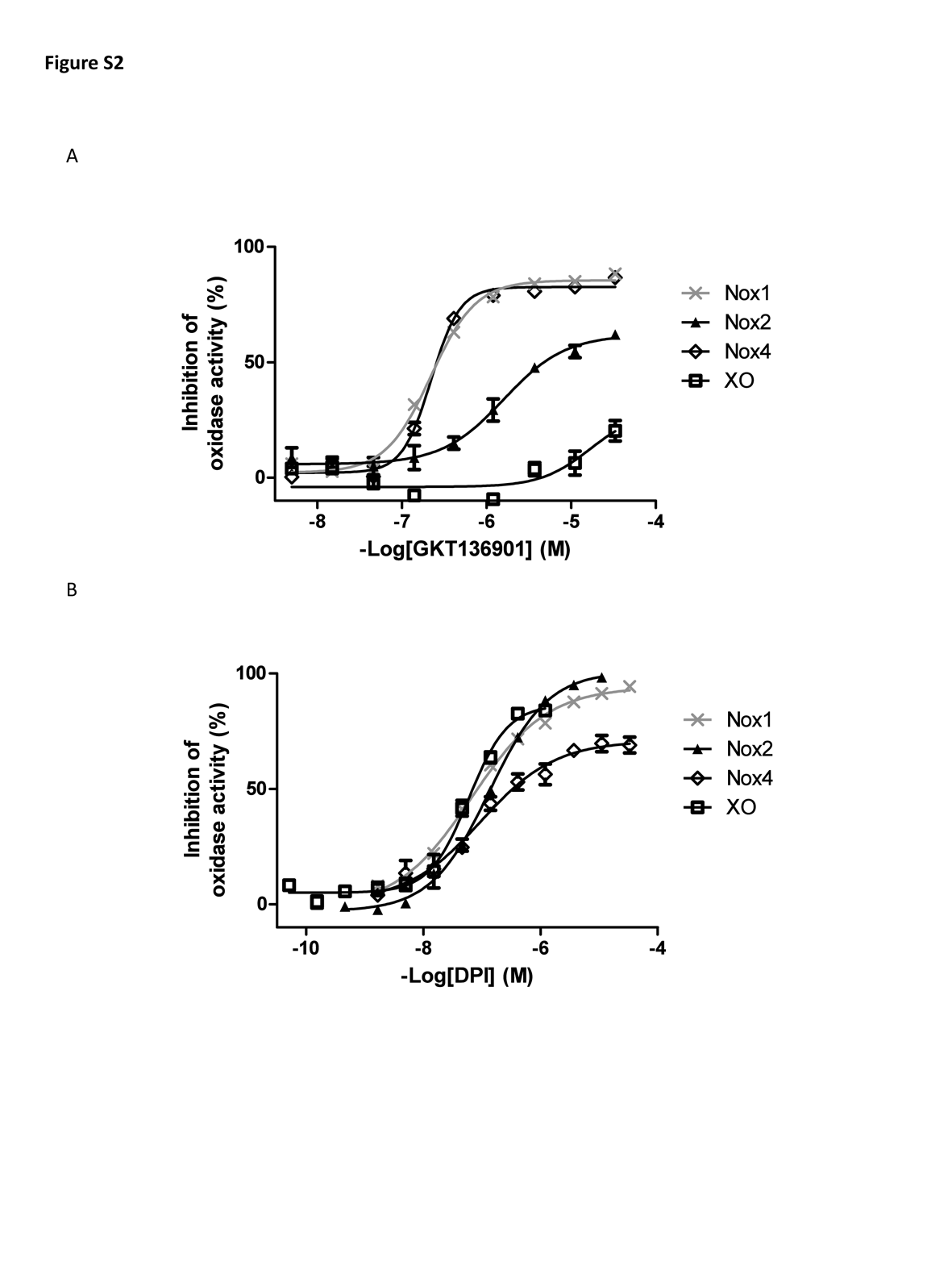

Supplement: Figure S2 — Inhibition of NOX-dependent ROS production by GKT136901 and DPI. a. Concentration-response curves of GKT136901 on NOX1 (x), NOX2 ▴), NOX4 (◊) and Xanthine Oxidase (XO) (□). b. Concentration-response curve of DPI on NOX1 (x), NOX2 (▴), NOX4 (◊) and Xanthine Oxidase (XO) (□) Results are from one experiment performed in triplicate, representative of four performed. Values are presented as means ± s.e.m. (2.02 MB TIF) [file pone.0014665.s003.tif]

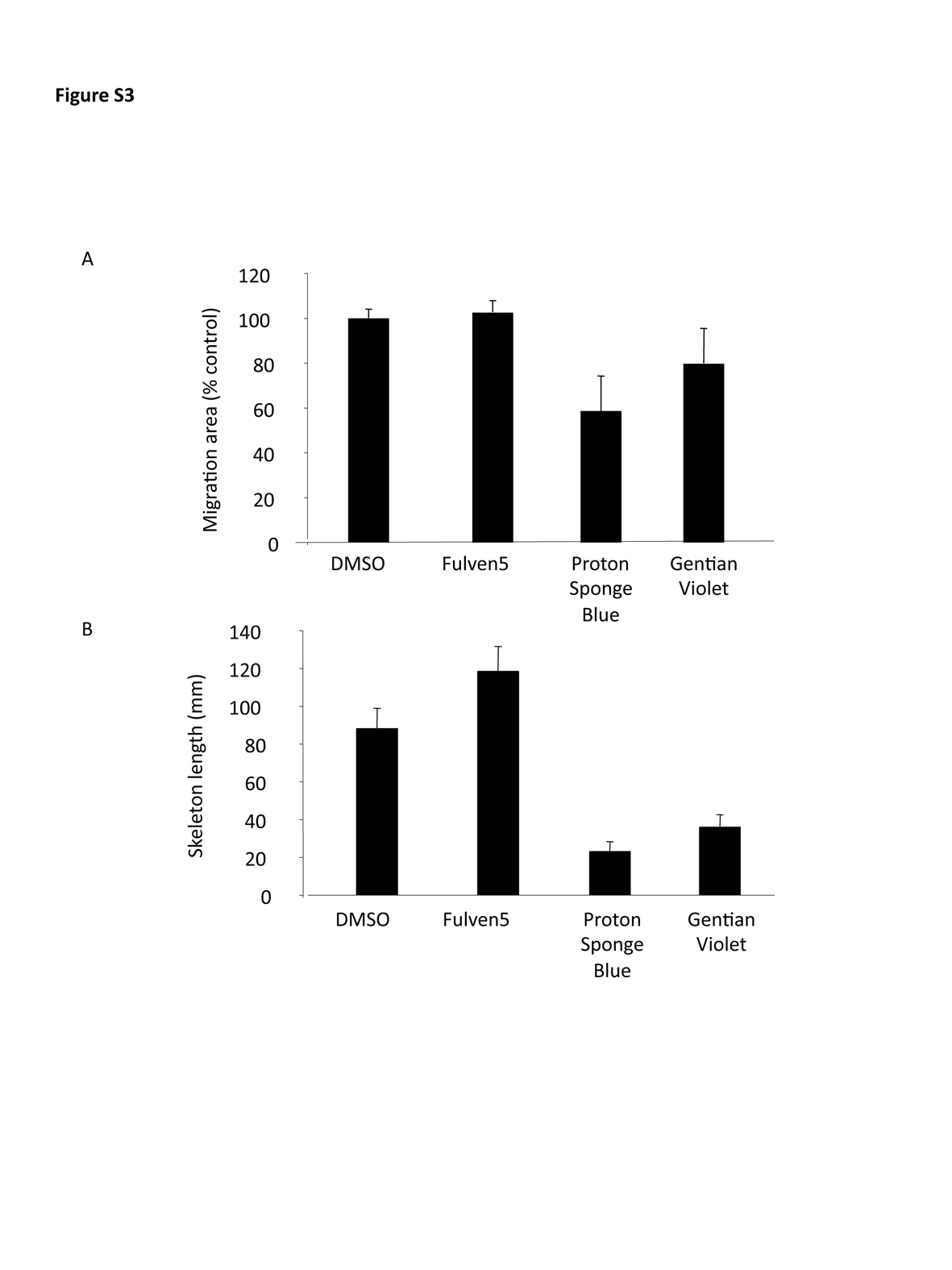

Supplement: Figure S3 — NOX dependant ROS blocking agents efficiently block endothelial cell migration and branching capacities. a. Migration of endothelial cells was analyzed by a wound-healing assay in presence of different inhibitors that block NADPH dependant ROS production. b. Tubular structure formation was measured by 3D culture using the mouse endothelial cell line in presence of different inhibitors that block NADPH dependant ROS production. Results are expressed in % of control ± s.e.m, n = 3. (5.89 MB TIF) [file pone.0014665.s004.tif]

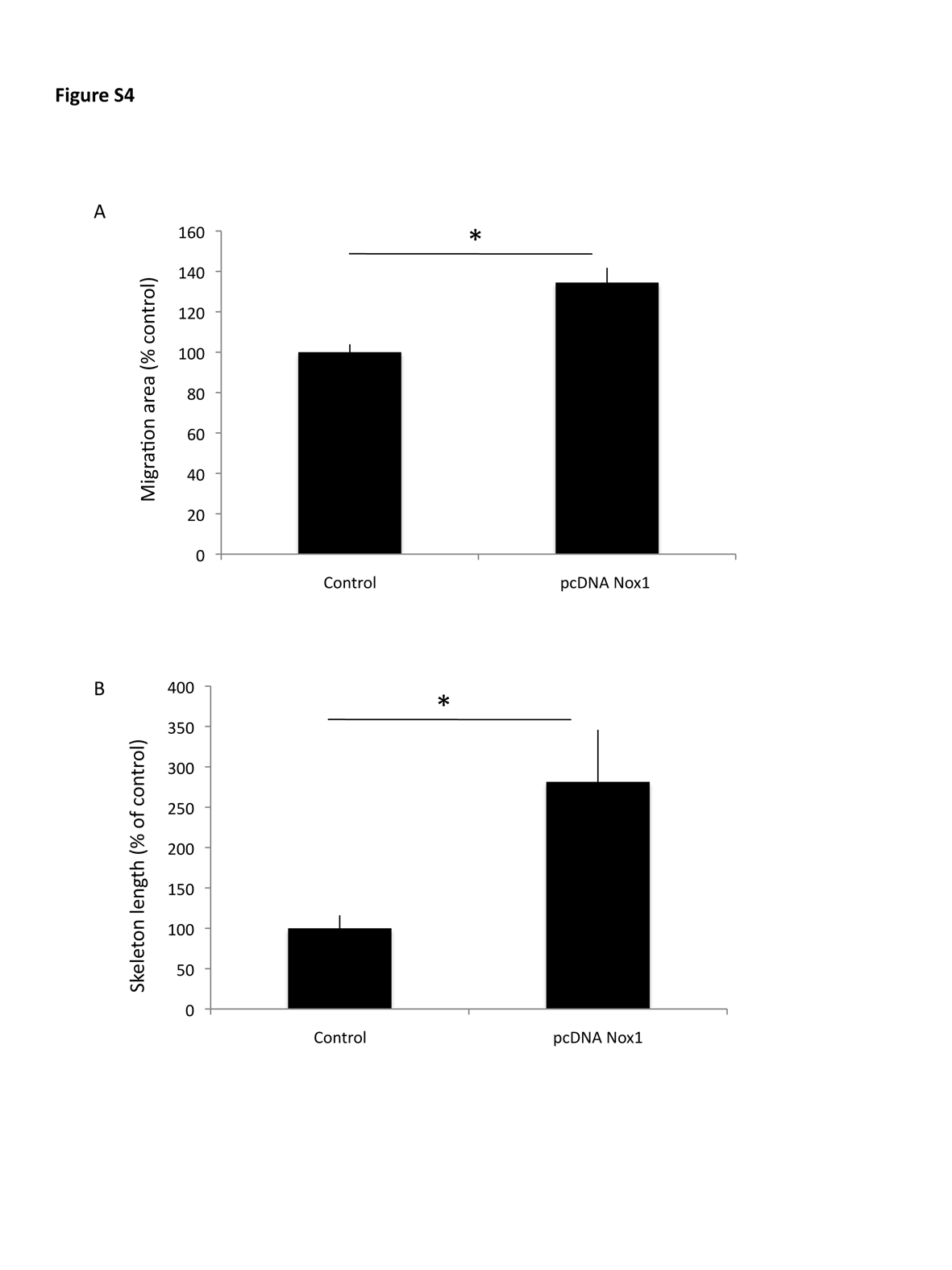

Supplement: Figure S4 — NOX1 over-expression enhances endothelial cell migration and tube-like structure formation. a. In vitro migration was analyzed by wound-healing assay using endothelioma cell lines transfected with NOX1 expressing vector. b. Tubular structure formation was measured by 3D culture of endothelioma cell lines transfected with NOX1 expressing vector. Results are expressed in % of control ± s.e.m. *p< 0.05 using Student's t-test. (5.88 MB TIF) [file pone.0014665.s005.tif]

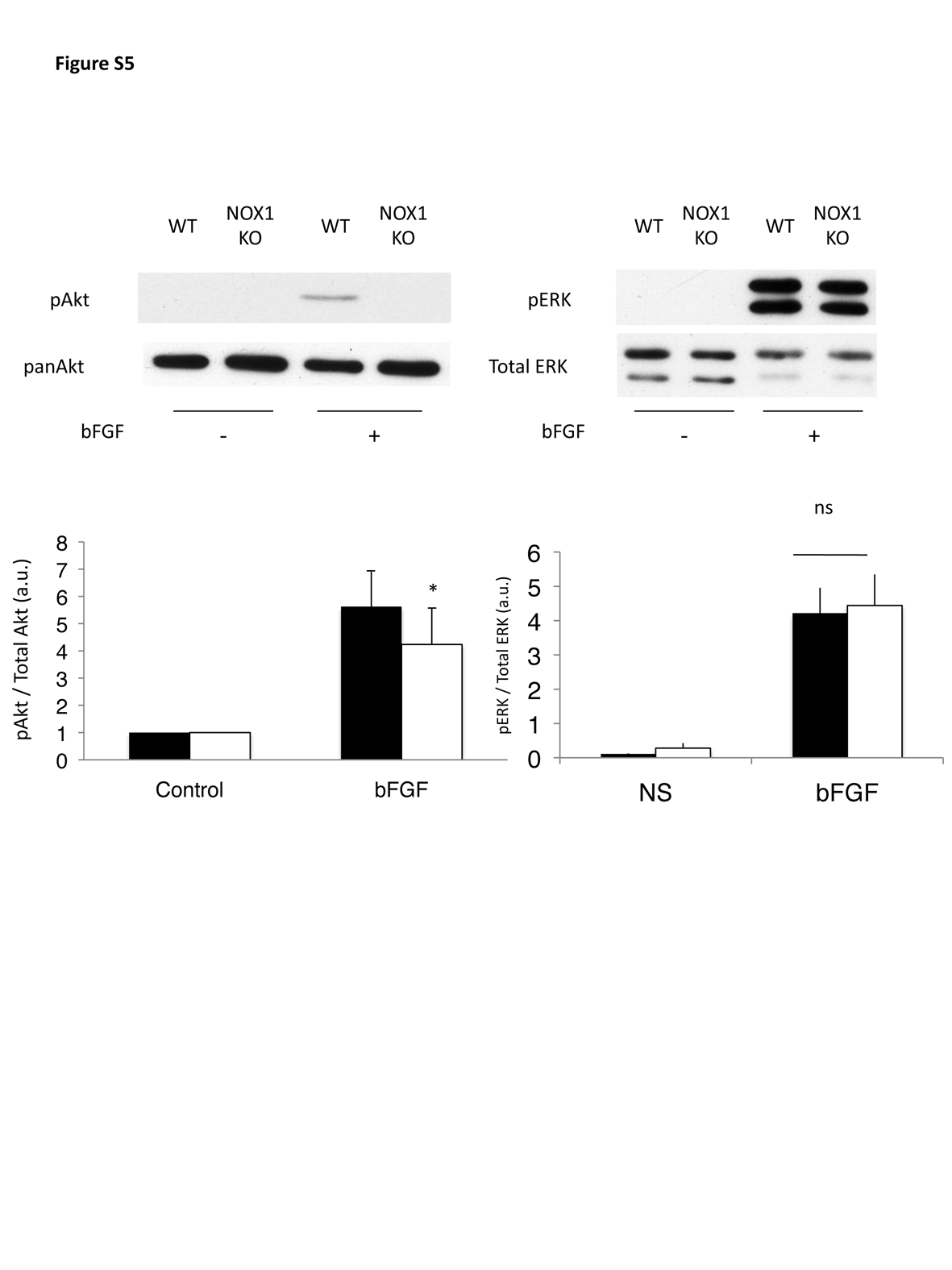

Supplement: Figure S5 — AKT but ERK 1/2 activation is affected by NOX1 deficiency. NOX1-deficient MLEC does not activate Akt after bFGF stimulation but present no difference in ERK1/2 activation. a. Western blot analysis of Akt phosphorylation in WT and NOX1-deficient MLEC after 10 min stimulation with 20 ng/ml of bFGF. The graph shows the abundance of phosphorylated Akt relative to total Akt ± s.e.m as determined by densitometry. n = 3. b. Western blot analysis of ERK1/2 phosphorylation in WT and NOX1-deficient MLEC stimulated for 10 min with 20 ng/ml of bFGF. The graph shows the abundance of phosphorylated ERK1/2 relative to total ERK1/2 ± s.e.m as determined by densitometry. n = 3. (6.13 MB TIF) [file pone.0014665.s006.tif]

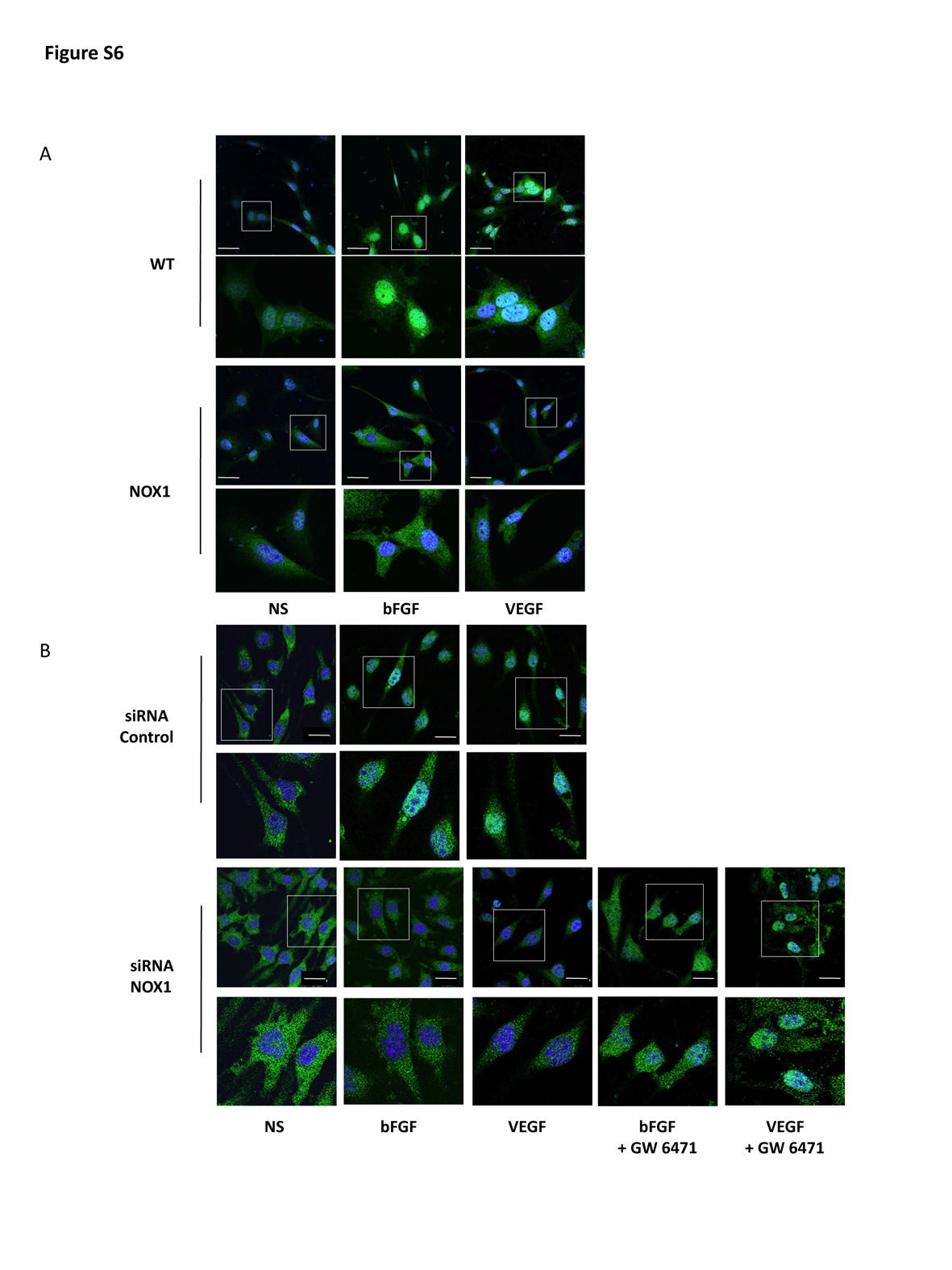

Supplement: Figure S6 — NF-κB nuclear translocation is inhibited in the absence of NOX1 and dependent on PPARα activation. VEGF or b-FGF stimulation of endothelial cells induced p65 NF-κB translocation into the nucleus. This nuclear translocation is not observed in NOX1-deficient cells but restored by PPARα antagonist treatment (GW6471). Immunofluorescence, anti-p65 NF-κB of MLEC (a) and endothelioma cell lines (b) stimulated with VEGF or bFGF in presence or absence of GW6471 (10mM). NF-κB in green (Alexa 488), nuclei in blue (DAPI). Scale bar represent 20 mm. Images were acquired with a 40x/1.3 numeric aperture and analyzed using LSM510 confocal microscope (Carl Zeiss). (7.09 MB TIF) [file pone.0014665.s007.tif]

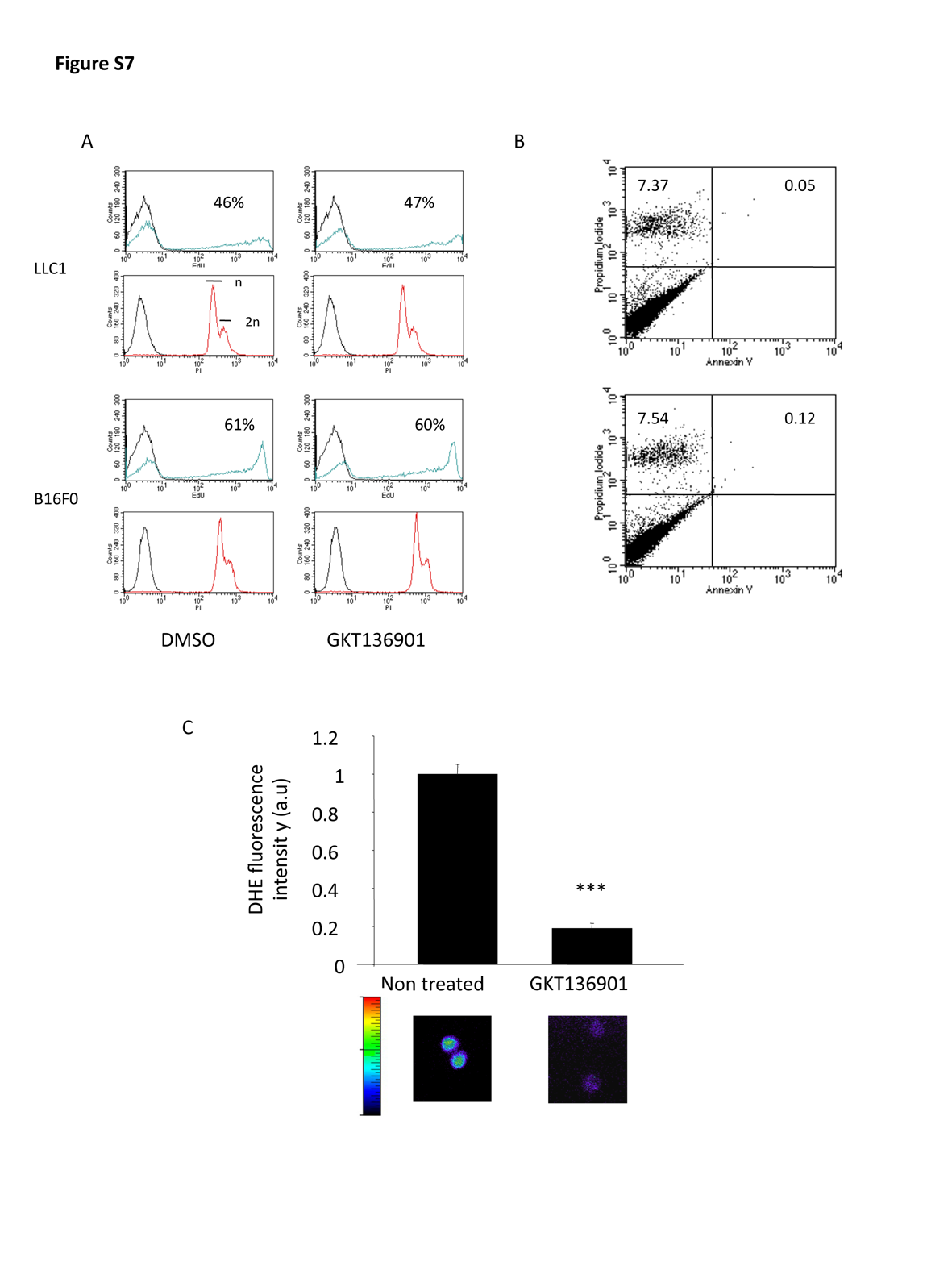

Supplement: Figure S7 — Effect of GKT 136901 on tumor cells. a. LLC1 and B16F0 cell proliferation was measured by EdU incorporation and propidium iodide staining of DNA content, 24h after incubation with 10 μM of GKT136901. b. LLC1 apoptosis was measured by AnnexinV/PI staining after 24h of incubation with 10 μM of GKT136901. c. ROS levels produced by LLC1 are inhibited by GKT136901. ROS production was quantified by DHE substrate 1h after incubation with 10 μM of the inhibitor. *** p<0.001 (student t-test). (6.11 MB TIF) [file pone.0014665.s008.tif]

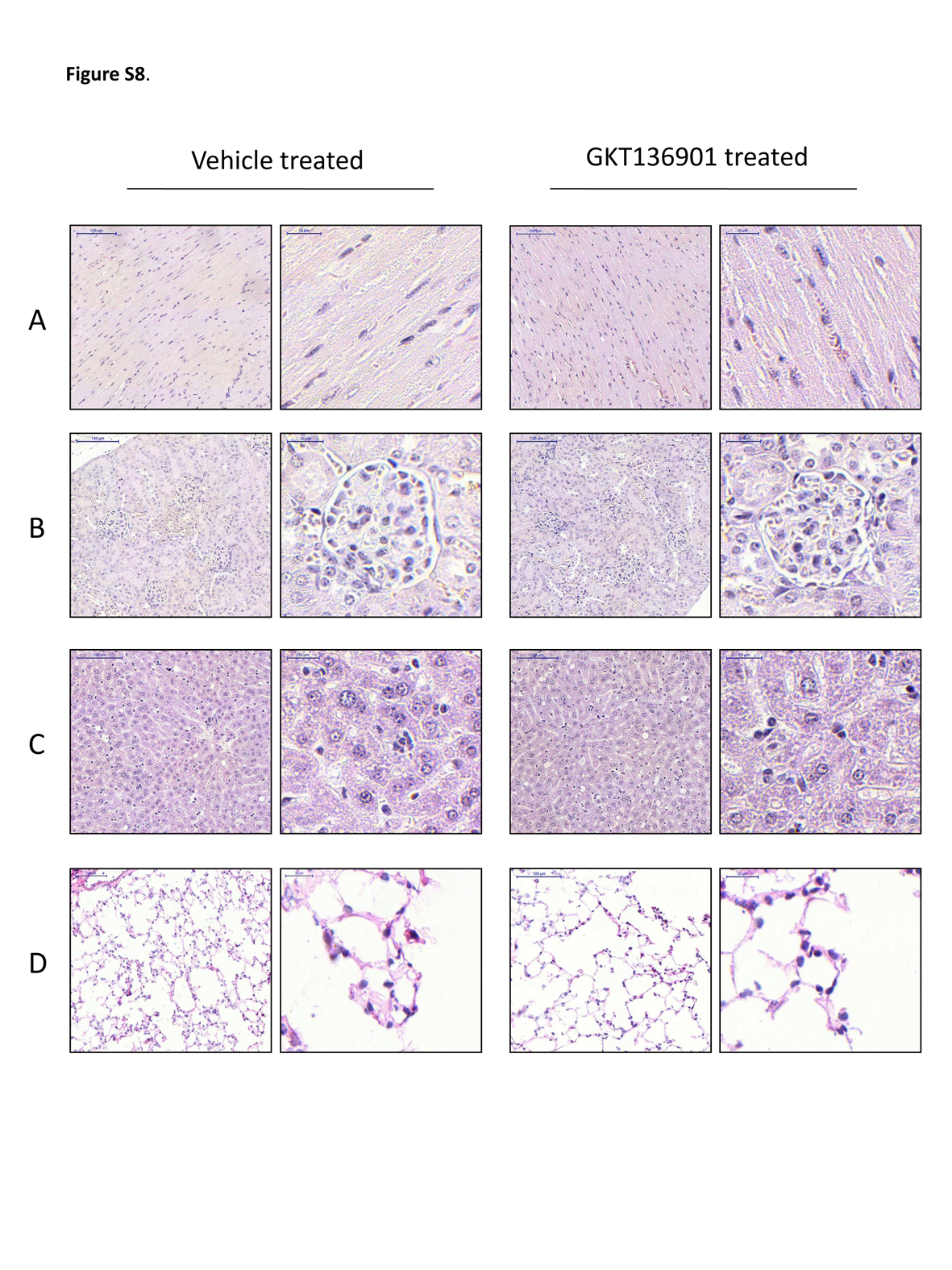

Supplement: Figure S8 — Non toxic effect GKT 136901 on mice organs. Heart (a), Kidney (b), Liver (c) and Lung (d) of mice treated orally with vehicle or with vehicle plus GKT136901 inhibitor at 40 mg/kg per day during 8 days, stained by Hematoxilin/Eosin. Scale bars represent 100 μm on the full picture and 20 μm on the zoom. Images were acquired with a 20x/0.8 numeric aperture and analyzed using Mirax (Carl Zeiss). (8.38 MB TIF) [file pone.0014665.s009.tif]
