# Supplementary material for: Targeting Vascular NADPH Oxidase 1 Blocks Tumor Angiogenesis through a PPARα Mediated Mechanism
Source: PLoS One. 2011 Feb 7;6(2):e14665. doi: 10.1371/journal.pone.0014665 (PMC3034713; doi:10.1371/journal.pone.0014665)
Supplement: Materials and Methods S1 — Real-time PCR primer sequence list. (0.05 MB DOC) [file pone.0014665.s010.doc]

| **Gene** | **Primer Forward** | **Primer Reverse** |
| --- | --- | --- |
| mu_nox1 | CAG TTA TTC ATA TCA TTG CAC ACC TAT TT | CAG AAG CGA GAG ATC CAT CCA |
| mu_nox2 | CAG GAA CCT CAC TTT CCA TAA GAT | AAC GTT GAA GAG ATG TGC AAT TGT |
| mu_nox4 | CCG GAC AGT CCT GGC TTA TCT | TGC TTT TAT CCA ACA ATC TTC TTG TT |
| mu_ppar-alpha | CCT CAG GGT ACC ACT ACG GAG T | GCC GAA TAG TTC GCC GAA |
| mu_ppar-gamma | TTC CAC TAT GGA GTT CAT GCT TGT | TCC GGC AGT TAA GAT CAC ACC TA |
| mu_tubulin | GCA GTG CGG CAA CCA GAT | AGT GGG ATC AAT GCC ATG CT |
| hu_nox1 | GAA GTG GGG CAG TAT ATC TTT GTT A | CTT CTA TCT TGA AAT CCA TCT GGT ACA AAT TC |
| hu_nox2 | TGG AGT GGC ACC CTT TTA CAC, | CCA CAA GCA TTG AAC AGC CC |
| hu_nox4 | CAG GAG GGC TGC TGA AGT ATC AA | TAT CCG GAG CAA TAA GCC AGT CA |
| hu_b2microglobulin | AAG ATG AGT ATG CCT GCC GTG | CGG CAT CTT CAA ACC TCC AT |
| mu_VEGF | AGCCGAGCTCATGGACGGGT | TGGCGGGCTCCTCTCCCTTC |
| mu_FGF2 | ATG GCG TCC GCG AGA AGA GC | ACC GGT TGG CAC ACA CTC CC |
| mu_Catalase | GGACAGTCGGGACCCAGCCA | ATTGGGTTCCCGCCTCCGGT |
| mu_GPX3 | TGGCTGAGCGCTTCGGACAC | AAGGCAGGATGCCCGGAGGA |
| mu_VCAM-1 | CCCGGATCTCAGGTGGCTGC | GCGTGGATTTGGCCCCCTCA |
| mu-MMP-2 | TTGGGCTGCCCCAGACAGGT | GTCCCACTTGGGCTTGCGGG |
| mu-MMP-9 | GGCCGCTCGGATGGTTACCG | TCGCGTCCACTCGGGTAGGG |
| mu-uPAR | CCA CAG CGA AAA GAC CAA CA | TGT CAG GCT GAT GAT CAT GGA |
| VE-Cadherin | GGA ACA ACC TTC CAG CTT CAC T | TGA ATA CCT CGT GCG AAA ACA C |

**Materials and methods S1:** Real-time PCR primer sequence list.
